# Comparison of the Effectiveness of Four Different Irrigation Solutions on Postoperative Sequelae in Patients Undergoing Lower Third Molar Surgery: A Prospective Study

**DOI:** 10.7759/cureus.50816

**Published:** 2023-12-20

**Authors:** Goutham Vijayakumar, Gidean A Sundaram, Santhosh P Kumar, Vinod K Krishna, Murugesan Krishnan

**Affiliations:** 1 Oral and Maxillofacial Surgery, Saveetha Dental College and Hospitals, Saveetha Institute of Medical and Technical Sciences, Saveetha University, Chennai, IND

**Keywords:** irrigation solution, normal saline, postoperative sequelae, mandibular third molar surgery, innovative practice, novel technique, povidone iodine, chlorhexidine, metronidazole

## Abstract

Introduction

Oral and maxillofacial surgeons frequently perform the removal of impacted mandibular third molars. The success of this surgical intervention depends on meticulous surgical technique and the use of appropriate irrigants to minimize complications in the postoperative period.

Aim

The aim of this study was to evaluate the efficacy of four different irrigation solutions (povidone-iodine, metronidazole, chlorhexidine gluconate (CHX), and normal saline) on postoperative sequelae like pain, trismus, swelling, and alveolar osteitis following surgical extraction of the impacted mandibular third molars.

Materials and methods

The current research was a randomized study carried out at Saveetha Dental College and Hospital in Chennai, India, from December 2022 to March 2023. The study population consisted of 112 participants who were referred to the Oral and Maxillofacial Surgery for the surgical removal of impacted mandibular third molars. The population was divided into four groups, with 28 in each group. They were categorized as A, B, C, and D based on the final irrigation solution used after surgical removal of the impacted teeth. In group A, patients received 0.5% povidone-iodine as the final irrigation solution; group B received 1% metronidazole; group C received 0.12% chlorhexidine gluconate (CHX); and group D received 0.9% normal saline. Patients were examined on the first and seventh postoperative days to assess pain, swelling, trismus, and alveolar osteitis. The results were analyzed with SPSS Statistics for Windows, Version 23.0 (Released 2015; IBM Corp., Armonk, New York, United States) software for Windows (Microsoft Corporation, Redmond, Washington, United States). A p-value less than 0.05 was considered statistically significant.

Results

Group B experienced significantly less pain than groups A, C, and D on the first and seventh postoperative days (p<0.05). The facial swelling was significantly less on the first and seventh postoperative day in group B compared to groups A, B, and D (p<0.05). There was no statistically significant variation observed in trismus (mouth opening) across the groups on both the first postoperative and seventh postoperative days. The presence of alveolar osteitis was seen in groups A, C, and D, but no instances were reported in group B.

Conclusion

It can be concluded that among the four irrigation solutions used in the lower third molar surgery, metronidazole irrigation solution yielded the best results in terms of less pain, swelling, and alveolar osteitis followed by chlorhexidine. There was no difference between povidone-iodine irrigation and normal saline irrigation on the postoperative sequelae. Postoperative trismus does not depend on the irrigation solution used in the third molar surgery.

## Introduction

Oral and maxillofacial surgeons frequently remove impacted mandibular third molars in dentistry; nevertheless, the procedure is not without any postoperative discomfort for the patient. These patients' discomforts and complaints are mostly due to pain, trismus, and swelling that occur post-operatively. Alveolar osteitis has also been reported in varying numbers after third molar removal, with incidence ranging from 25% to 30%. Alveolar osteitis is characterized by postoperative pain in and around the extraction site, which increases in severity between one and three days after the extraction, accompanied by a partial or total disintegrated blood clot within the alveolar socket with or without halitosis [1]. According to Birn, this could be the result of fibrinolysis subsequent to traumatic extraction or microorganisms that can cause wound infection [2]. Although alveolar osteitis is widely regarded as having a multifactorial origin, the following have been identified as the most frequent causes: difficulty and traumatic extraction, physical dislodgement of the clot, and oral contraceptives [3].

Microorganisms associated with dry sockets include Actinomyces viscosus, Streptococcus mutans, and Treponema denticola. Numerous studies have concentrated on lowering the intra-oral bacterial count to decrease the occurrence of alveolar osteitis [3,4]. It has been described in the past that the use of either topical or systemic antibiotics that inhibit these microorganisms in the extraction socket can reduce the occurrence of alveolar osteitis and promote wound healing [4]. Prostaglandins and other mediators generated from membrane phospholipids released after surgery cause pain, edema, and trismus. The difficulty of extraction also increases the magnitude of the pain, swelling, and trismus [5]. The most widely used irrigation solution during the guttering process in the surgical removal of an impacted tooth is normal saline. After surgery, the socket is irrigated with copious volumes of normal saline to eliminate any debris that could induce alveolar osteitis, pain, swelling, and trismus. Using antibiotic solutions as the final irrigation solution can also help prevent them.

The aim of this study was to evaluate the efficacy of four different irrigation solutions: povidone-iodine, metronidazole, chlorhexidine, and normal saline on postoperative sequelae (pain, trismus, swelling, and alveolar osteitis) following surgical extraction of the impacted mandibular third molars. The objectives of the study were to compare the four irrigation solutions in the reduction of pain and swelling after lower third molar surgery, to assess the efficacy of the irrigation solutions in the improvement of mouth opening postoperatively, and to evaluate the incidence of alveolar osteitis postoperatively.

## Materials and methods

Study design and setting

The current research was a prospective clinical study carried out at the Department of Oral and Maxillofacial Surgery, Saveetha Dental College and Hospital, Chennai, from December 2022 to March 2023. This was approved by the institutional Human Ethical Committee (IHEC/SDC/OMFS-2205/22/255).

Inclusion criteria

The participants were individuals requiring the surgical extraction of an impacted mandibular third molar who had a Pederson difficulty index score of 5-8 on a 10-point scale with no symptoms one week prior to surgery and were categorized by the American Society of Anesthesiologists (ASA) physical status classification system as ASA I or ASA II. Patients who provided consent for undergoing the treatment under the administration of local anesthesia and were not on any antibiotics or anti-inflammatory medications one week prior to the surgery were included in the study.

Exclusion criteria

Patients with ASA classification II or higher, preexisting infections, diabetes mellitus, uncontrolled hypertension, bone pathology, smoking habits, alcoholism, any neurological deficits, and allergies to chlorhexidine, metronidazole, or povidone-iodine, and pregnant or lactating women were excluded from the study.

Surgical procedure

The study population consisted of 112 participants who were referred to the Oral and Maxillofacial Surgery Department and were divided into four groups with 28 members in each group. Informed consent was obtained from all the participants before the procedure. Before any surgical intervention, a dental orthopantomogram was taken for each participant in the study to assess the impacted teeth and rule out any underlying diseases. Patients were reviewed on the first and seventh postoperative days. 100 patients were randomly allocated into four groups. They were categorized as A, B, C, and D based on the final irrigant used after surgical removal of the impacted teeth. In group A, patients received 0.5% povidone-iodine as the final irrigant; group B received 1% metronidazole; group C received 0.12% chlorhexidine gluconate (CHX); and group D received 0.9% normal saline. 

**Figure 1:**
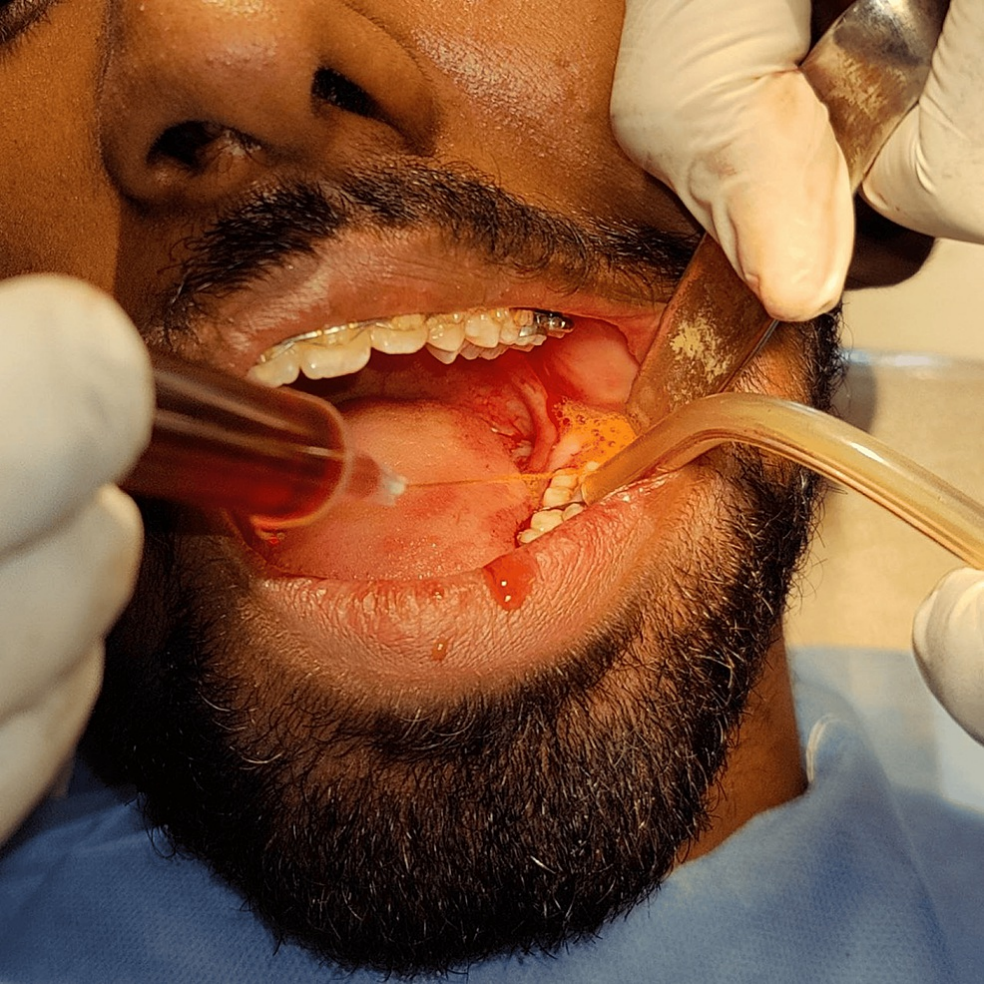
Final wash of the surgically removed lower third molar socket with povidone-iodine irrigation solution

Under aseptic precautions, all patients received inferior alveolar, lingual, and long buccal nerve blocks. Two percent lignocaine with 1:80,000 adrenaline was used as a standard local anesthetic for all the participants. A full-thickness triangular mucoperiosteal flap was elevated, and bone guttering was performed using a tungsten carbide bur. Normal saline (0.9%) was used as the standard irrigation solution during guttering in all the groups. The tooth was sectioned and removed. Post-extraction final rinsing of the socket was done with 20 ml of the respective irrigation solution in each of the four groups. Figure 1 depicts the final rinsing of the socket with povidone-iodine irrigation solution post-extraction. Primary closure of the surgical wound was done using a 3-0 black braided silk suture. Patients were prescribed the same oral antibiotics (amoxicillin) and analgesics (aceclofenac and paracetamol combination) in all the groups.

Assessment

Patients were examined on the first and seventh postoperative days to assess pain, swelling, trismus, and alveolar osteitis. Pain was assessed by the Visual Analogue Scale (VAS) on a scale ranging from 0 to 10 with 0 indicating no pain, 5 indicating moderate pain, and 10 indicating worst pain. The evaluation of trismus involved assessing the maximum mouth opening by measuring the inter-incisal distance. For measuring swelling, the three-line Laskin method of facial swelling measurement was used. Alveolar osteitis was evaluated based on the existence of pain and the blood clot dislodgement irrespective of the presence or absence of halitosis for three days following the surgical procedure.

Statistical analysis

The results were analyzed with SPSS Statistics for Windows, Version 23.0 (Released 2015; IBM Corp., Armonk, New York, United States) software for Windows (Microsoft Corporation, Redmond, Washington, United States). Using the Shapiro-Wilk test, the normality of the data was determined. Frequency and percentage were determined to assess the incidence of alveolar osteitis. The difference in swelling and trismus within the groups across the timeline was measured by repeated measures analysis of variance (ANOVA), and the difference in swelling and trismus between the groups was measured by one-way ANOVA. The Friedman test was used to assess the difference in pain within the groups across the timeline, and the Kruskal-Wallis test was used to assess the difference in pain between the groups. A p-value less than 0.05 was considered statistically significant.

## Results

**Figure 2:**
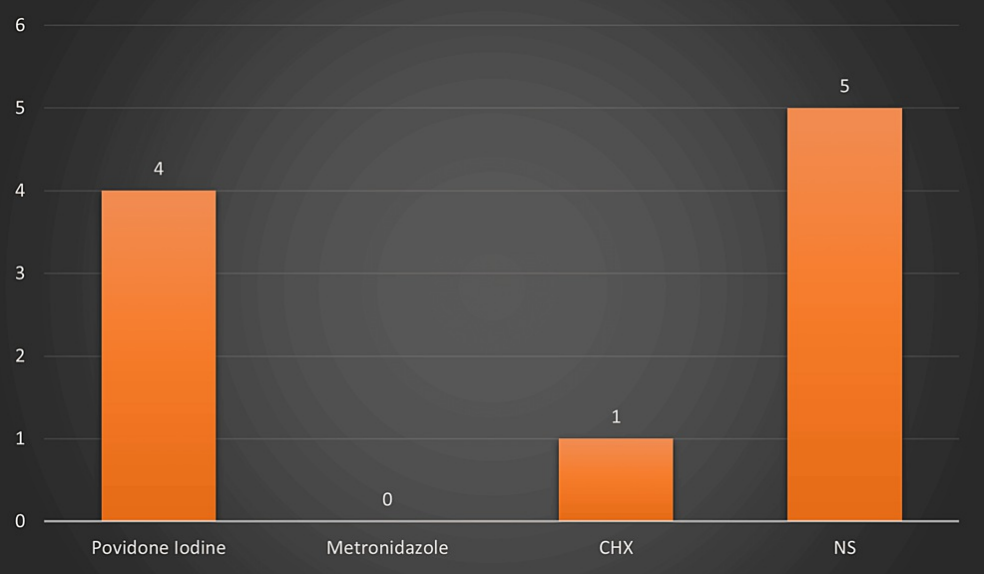
Incidence of alveolar osteitis between the groups CHX: Chlorhexidine; NS: Normal saline

The study population consisted of 112 participants who underwent surgical removal of impacted mandibular third molars. The population was divided into four groups: A, B, C, and D, with 28 participants in each group. The incidence of alveolar osteitis postoperatively between the groups is depicted in Figure 2. The highest incidence occurred in patients in whom normal saline was used, followed by the povidone-iodine group. There was no incidence of dry socket in the metronidazole irrigation solution group.

The normality tests revealed that the distribution of swelling and trismus data was normal; hence, parametric tests were used. For pain data, non-parametric tests were used. The difference in mean pain scores within the groups decreased from preoperative day to seventh postoperative day in all the groups and the results were statistically significant (p<0.05) with lowest scores seen in the metronidazole group followed by the chlorhexidine group. The difference in mean pain scores between the groups preoperatively was not statistically significant (p=0.568). The difference in mean pain scores between the groups on the first postoperative day was statistically significant with the least pain exhibited in metronidazole group and the maximum pain present in the normal saline group (p=0.003). The difference in mean pain scores between the groups on seventh postoperative day was statistically significant with the least pain exhibited in metronidazole group and maximum pain present in the normal saline group (p=0.013) (Table 1).

**Table 1:** Comparison of pain between the groups Data is represented as mean ± standard deviation (SD), p<0.05 * - Statistically significant, Within group comparison - Friedman test, Between groups comparison - Kruskal-Wallis test CHX: Chlorhexidine

Mean pain scores at different timelines (mean ± SD)	Povidone-iodine	Metronidazole	CHX	Normal saline	p-value
Preoperative	7±1.48	8.55±0.82	7.18±1.4	7.64±1.69	0.568
Postoperative day one	6.09±1.64	4.64±1.5	6.09±1.44	7.36±1.69	0.003*
Postoperative day seven	2.27±1.32	1.09±0.94	1.55±1.03	2.82±1.68	0.013*
p-value	0.001*	0.001*	0.039*	0.045*	

The mean mouth opening scores within the groups increased (less trismus) from preoperative day to seventh postoperative day in all the groups and the results were statistically significant (p<0.05). The difference in mean trismus scores between the groups, preoperatively (p=1.012), on the first postoperative day (p=0.954), and on the seventh postoperative day (p=0.922), was not statistically significant (Table 2).

**Table 2:** Comparison of trismus between the groups Data is represented as mean ± standard deviation (SD), p<0.05 * - Statistically significant, Within group comparison - Repeated measures analysis of variance test, Between groups comparison - One-way analysis of variance test CHX: Chlorhexidine

Mean mouth opening scores at different timelines (mean ± SD)	Povidone-iodine	Metronidazole	CHX	Normal saline	p-value
Preoperative	19.36±5.44	17.18±4.79	16.18±5.01	18.63±5.81	1.012
Postoperative day 1	21.36±6.32	22.27±6.48	20.72±6.54	21.36±6.2	0.954
Postoperative day 7	46.18±7.11	47.18±6.61	45.18±6.61	46.18±6.61	0.922
p-value	0.008*	0.001*	0.001*	0.001*	

The difference in mean swelling scores within the groups increased from the preoperative day to the first postoperative day in all the groups. The difference in mean swelling scores within the groups decreased from the first postoperative day to the seventh postoperative day in all the groups with the lowest swelling scores seen in the metronidazole group (p=0.001) followed by the chlorhexidine group (p=0.001) and the results were statistically significant. The decrease in mean swelling scores in the povidone-iodine group and normal saline groups was not statistically significant (p>0.05). The difference in mean swelling scores between the groups preoperatively was not statistically significant (p=0.846). The difference in mean swelling scores between the groups on the first postoperative day was statistically significant with the least swelling exhibited in the metronidazole group and maximum swelling present in the normal saline group (p=0.001). The difference in mean swelling scores between the groups on the seventh postoperative day was statistically significant (p=0.001) with the least swelling exhibited in the metronidazole group and maximum swelling present in the normal saline group (Table 3).

**Table 3:** Comparison of swelling between the groups Data is represented as mean ± standard deviation (SD), p<0.05 * - Statistically significant, Within group comparison - Repeated measures analysis of variance test, Between groups comparison - One-way analysis of variance test CHX: Chlorhexidine

Mean swelling scores at different timelines (mean ± SD)	Povidone-iodine	Metronidazole	CHX	Normal saline	p-value
Preoperative	103.23±1.91	105.71±4.26	109.66±7.13	113.62±2.08	0.846
Postoperative day 1	108.43±1.41	110.45±4.42	117.23±5.53	117.54±1.48	0.001*
Postoperative day 7	103.24±1.97	98.59±2.63	101.25±2.51	112.76±2.55	0.001*
p-value	0.074	0.001*	0.001*	0.052	

## Discussion

For the guttering process of impacted tooth removal, an ideal irrigation solution should be readily available, which must be isotonic, biocompatible, non-toxic, and cost-effective [6,7]. The process of irrigating during surgery helps clear the surgical site of blood, bony debris, and foreign objects, leading to better visibility and reduced bacterial presence, which in turn supports better healing [8]. Chlorhexidine is a cationic biguanide with broad-spectrum coverage against both Gram-positive and Gram-negative bacteria. Chlorhexidine has bacteriostatic properties at low concentrations (0.02%-0.06%), bactericidal properties at high concentrations (>0.12%), antifungal properties, and some antiviral properties. Its effect is not hampered by biological fluids such as blood, and it works for up to 48 hours after application [9]. It acts by penetrating the bacterial cell membrane, causing cytoplasmic leakage at lower concentrations and precipitating nucleic acid at higher concentrations. Studies by Ghosh et al. [9] and Reddy et al. [10] have proven that the use of 0.12% chlorhexidine gluconate as an irrigation solution or mouth rinse postoperatively significantly reduces the incidence of dry socket after the removal of impacted third molars. Alveolar osteitis, also known as dry socket, occurs due to the inflammation of the alveolar bone involving either the maxilla or mandible. Previous research has demonstrated that chlorhexidine is more efficacious than povidone-iodine at reducing postoperative pain and alveolar osteitis following extraction of lower third molars [10].

Povidone iodine is an iodine compound that possesses rapid and broad-spectrum antimicrobial properties against bacteria, fungi, and viruses [11]. It releases free iodine that penetrates the microorganism, causing oxidation of the amino acid, nucleic acid, and cell membrane, which ultimately leads to cell death. Using it as an irrigation solution during maxillofacial surgery decreases oral bacterial counts considerably for up to an hour following the treatment [12].

Comparative studies have been conducted with normal saline, chlorhexidine, and povidone-iodine as irrigation solutions for wisdom tooth removal which assessed healing and postoperative complications and found no significant differences between the groups [11-13]. According to the research by Jadhao et al., chlorhexidine was more effective than povidone-iodine in the reduction of postoperative pain, alveolar osteitis, trismus, and swelling [14].

Metronidazole is a nitroimidazole with activity against most Gram-negative and Gram-positive anaerobic bacteria. Its antibacterial effect is dose-dependent, with its post-antibiotic effect lasting up to three hours [15]. Bergdahl et al. studied its role in the prevention of alveolar osteitis by administering a single oral 1600 mg tablet of metronidazole before extraction and found no statistical difference between the metronidazole and placebo groups [16]. Haghighat et al. compared the use of 1% metronidazole to normal saline as an irrigation solution in third molar removal and found no statistically significant difference between the two groups [17].

In all the previous studies [10,13], the solution has been either used as a continuous irrigation solution or as a post-operative irrigation solution. These drugs were also prescribed as tablets and their effects on postoperative sequelae in lower third molar surgery were compared in research studies [18,19]. Our study evaluated the action of these four irrigation solutions when used as a final rinsing solution in the surgical removal of the impacted mandibular third molars. Since normal saline irrigation solution is commonly used, it was considered as a standard solution for comparison with other irrigation solutions. Although alveolar osteitis is multifactorial, this study only aims to highlight the microbial cause and the influence of antibacterial irrigants in the sequela of mandibular third molar surgery.

In summary, after evaluating the irrigation solutions (metronidazole, chlorhexidine, povidone-iodine, and normal saline) in the context of impacted mandibular third molar surgery, it was observed that metronidazole irrigation yielded the best results in terms of less pain, swelling, and alveolar osteitis followed by chlorhexidine. Postoperative trismus does not depend on the irrigation solution being used.

Limitations of the study

Further randomized clinical trials with larger sample sizes are necessary to assess the findings observed in this study.

## Conclusions

It can be concluded that after evaluating the irrigation solutions (metronidazole, chlorhexidine gluconate, povidone-iodine, and normal saline) in the context of impacted mandibular third molar surgery, it was observed that metronidazole irrigation yielded the best results in terms of less pain, swelling, and alveolar osteitis followed by chlorhexidine gluconate. Postoperative trismus does not depend on the irrigation solution being used. There was no difference between povidone-iodine irrigation and normal saline irrigation in the postoperative sequelae. Thus, metronidazole irrigation solution can be administered in mandibular third molar surgeries for favorable post-surgical outcomes. 
